# Economic impacts of biodiesel policy in Indonesia: a computable general equilibrium approach

**DOI:** 10.1186/s40008-022-00281-9

**Published:** 2022-11-09

**Authors:** Sahara, Ahmad Dermawan, Syarifah Amaliah, Tony Irawan, Salsa Dilla

**Affiliations:** 1 Department of Economics, Faculty of Economics and Management, IPB University, Bogor, Indonesia; 2 Center for International Forestry Research-World Agroforestry (CIFOR-ICRAF), Bogor, Indonesia; 3 Public Administration and Policy Group, Wageningen University, Wageningen, Netherlands

**Keywords:** Economic impact, Biodiesel, Computable general equilibrium, Palm oil, Trade

## Abstract

The Government of Indonesia has been promoting the advancement of the biodiesel sector to fulfill its commitment to support clean energy, energy security, and rural development. This paper examines the economic impact of the biodiesel sector using a computable general equilibrium model. Besides analyzing the impacts on the national macroeconomic conditions, other sectors, and household incomes, our model has also included a regional block to capture the impact of the biodiesel mandate on regional growth. Two simulations were performed: (1) fulfillment of the 30% biodiesel blending target (B30 mandate), and (2) Simulation 1 combined with the European Union's biodiesel trade ban resulting in an export reduction of 5.18%. The results show that the two simulations provide positive impacts on macroeconomic variables, including real gross domestic product and real wages. However, the B30 mandate and the combined effect of the EU trade ban still yield an inflationary effect in the short term. They also potentially reduce the production of several agricultural products—such as sugarcane, fruits, vegetables, and soybeans—leading to an increase in food prices. The policy implications highlight that the current B30 mandate and EU ban cannot automatically improve the fuel trade balance.

## Introduction

Biodiesel is a promising renewable energy source for Indonesia, a nation known for its renewable resource potential for biofuels. For the past few years, the focus on biodiesel has greatly increased due to the reduction in fossil-fuel oil production in Indonesia, crude oil price trends at the global level, and the country's trade deficit in the oil and gas sector. According to the Ministry of Energy and Mineral Resources (MEMR) (2021), crude oil production in Indonesia was about 745.14 thousand barrels of oil per day (BOPD) in 2019 and fell to 719.99 BOPD in 2020 (a decline of 3.37%). After experiencing demand and supply shocks due to Covid-19 since 2020, the economic recovery in 2021 increased the price of crude oil to about USD 70 per barrel from about USD 20 per barrel in mid-2020 (World Energy Outlook 2021). Rising global oil prices impact the Indonesian trade balance because the country has become an oil-importing country as there has not been any significant increase in oil sector investment and production capacity. From January 2017 to September 2018, Indonesian oil and gas imports rose 27.14%, resulting in a trade deficit of USD 9.4 billion in the oil and gas sector (MT 2018). In 2019 and 2020, the trade deficits in the sector still amounted to about USD 5.4 billion and USD 9 billion, respectively.

Based on these figures, Indonesia is taking a massive risk if it continues to depend on fossil fuels, which have been used excessively and rapidly depleted. The international community's concerns about the urgent need to reduce greenhouse gas emissions are increasing pressure on governments in many countries, including Indonesia, to develop renewable energy sources. Attention is currently focused on biodiesel since Indonesia has strong production potential in this sector, particularly from palm oil. Indonesia is the largest palm oil producer in the world, contributing about 50% to the total global supply (Sahara et al. 2017). The country increased its palm oil production from 31.07 million tons in 2015 to 42.9 million tons in 2018 (Indonesia Directorate General of Estates 2019). It is predicted to boost its output to 49.71 million tons in 2021 (Ministry of Agriculture 2021).

In response to these challenges, i.e., the growing scarcity of fossil fuels, price volatility of fossil fuels, and increasing trade deficit of the oil sector, the Government of Indonesia (GoI) has introduced biodiesel development policies. The mandate also aims to increase the added value of palm oil, stabilize crude palm oil production at the domestic level, and improve farmers' income and rural development. Since its introduction in 2015, the mandate has been implemented primarily in the public service obligation (PSO) category for transportation—which accounts for 90% of national oil use—and electricity generation. Recently, the mandate was extended. The recent B20 blending mandate for all sectors of the economy covers subsidized diesel oil and non-subsidized diesel oil (USDA 2018). The B20 mandate comprises a mix of 20% vegetable oil from crude palm oil and diesel oil to produce biodiesel. The initiative was implemented in September 2018, extending a prior regulation signed in 2016, when B20 applied only to subsidized diesel oil. The GoI also aimed to accelerate the implementation of a B30 mandate to 2019, ahead of its original start date in 2020. The mandate requires the blending composition of biodiesels to include 30% crude palm oil (B30) and 70% diesel oil (Siantar and Nugraha 2018). In 2020, the total production of biodiesel (including the production through the biodiesel program) reached about 8.13 million kiloliters, while consumption amounted to about 9.2 million kiloliters (MEMR 2021). To support the biodiesel program, the GoI provided subsidies by introducing a palm oil export levy, which is collected by a designated public service agency called the Estate Crop Fund for Palm Oil (BPDPKS). These levies will be used to subsidize biodiesel once the diesel oil price index is more economical than the biodiesel price index. As of May 2022, the GoI imposes an export levy of USD 200/metric ton for crude palm oil (Ministry of Finance 2022).

The implementation of the B30 mandate faces several challenges. First, the increase in the price of Crude Palm Oil (CPO), particularly in 2021, highlights the sustainability of feedstock for the biodiesel program since it will be more profitable to export crude palm oil than to fulfill the biodiesel program. Second, funding the program has required a large amount of government subsidies. For example, in 2021, GoI subsidies for the mandatory 30% biodiesel (B30) program reached IDR 44 trillion (Wibowo 2021). Third, the heated issue of "food versus fuel" indicates that increased biodiesel production may lead to stronger land competition among actors, leading to higher food prices (Kretschmer and Peterson 2010). Fourth, there is the challenge of exporting biodiesel since the United States has implemented anti-dumping and countervailing duties on Indonesian biodiesel. At the same time, the European Union's Renewable Energy Directive Recast (RED II) is likely to cap crop-based biodiesels by 2030. This will reduce the demand from the EU biodiesel industry for palm oil (USDA 2018).

Considering these four challenges, it is timely to offer a more comprehensive understanding of the economy-wide impacts of enhanced biodiesel production (B30) on the Indonesian economy at the macro- and micro-levels and on regional growth. This broad scope is essential since biodiesel production impacts not only the palm oil and oil sectors but also other sectors of the economy. As such, a computable general equilibrium (CGE) model is employed in the study. We have updated the database for the Indonesian CGE by using the latest input–output (I–O) table published by Statistics Indonesia in 2020. To capture the regional impact, we also included the latest interregional input–output (IRIO) published by Statistics Indonesia in 2021. By having the IRIO, the impact of the B30 mandate on regional growth (at a provincial level) can be captured.

This paper is organized into five parts, including this introduction. The second section describes the impact of biodiesel development in several countries based on existing literature. The third section provides the main data and methods used in this paper. The fourth section describes the findings, and the fifth consolidates this paper's main conclusions and implications.

## Literature review

Policies to promote the production and use of biofuels (biodiesel and bioethanol) have been implemented since the early 2000s, both in developed and developing countries. According to Gunatilake et al. (2011a), adopting biofuel as a renewable energy source offers opportunities for climate change mitigation and greater energy security for many countries. According to Khan et al. (2021), biofuels are arguably a potential renewable energy source in the transportation industry. The issues of climate stabilization and rapid innovation to reduce greenhouse gas (GHG) emissions have also stimulated a new agribusiness energy industry, particularly in high-income economies with more energy-intensive expenditure (Berndes et al. 2003; Farrell et al. 2006). Using biodiesel could reduce a country's dependency on diesel fuel imports, cutting hard currency spending significantly (Adolphe 2007).

Studies on biofuel use and its economic and environmental effects have been conducted in developed and developing countries. Many papers have used CGE models to analyze the impacts of biofuel policy implementation on countries or regions, such as the United States, the EU, Brazil, India, and Tanzania (Arndt et al. 2012; Elobeid et al. 2006; Gunatilake et al. 2011b; Gohin 2008; Tyner and Taheripour 2008).

Some studies show a positive impact of biofuel production on economic performance. Gunatilake et al. (2011a) reported that, in India, biodiesel could enhance energy security, generate significant employment and achieve inclusive growth, without adverse impacts on other sectors of the economy. Biodiesel can be used as a transport fuel substitute that can be produced in ways that fully utilize marginal agricultural resources and improve rural livelihoods. Gunatilake et al. (2011b) further examined the impacts of biodiesel expansion on household welfare, other sectors of the economy, carbon emissions, rural development, and employment generation in India. The results showed that expanding biodiesel production to meet the national target improves welfare. The sector can generate 0.7% to 1.0% one-time incremental growth with significant employment and income generation in rural areas.

Salleh et al. (2020) found that establishing a comprehensive and inclusive national bioenergy policy will lead to a sustainable future of renewable energy development in Malaysia. The study suggested that future emphasis should shift from solar power to biomass and biogas. It also investigates the strategies that could be adapted to promote the diversification of renewable energy resources. The paper proposed a new national bioenergy policy through four essential programs: (i) enhanced bioenergy conversion efficiency and waste management; (ii) biomass co-firing in coal power plants; (iii) conversion of biogas to biomethane and bio-compressed natural gas (bio-CNG), and (iv) large-scale biomass power plants.

Altenburg et al. (2009) reported that biodiesel production could create additional sources of income for India's rural population. This could be done by intensifying land use while greening the countryside. Proponents of biodiesel point to the potential of oilseeds as a substitute for fossil fuels, underlining their ability to reduce India's energy dependency and bring down GHG emissions. The developmental effects differ between the many ways of organizing biodiesel value chains. They differ between the three categories of value chain organization due to the various objectives of their respective main actors: (i) achieving social welfare and environmental protection in the case of the government; (ii) generating additional income in the case of farmers, and (iii) maximizing productivity and returns on investment in the case of corporate investors. Whether these effects materialize depends on policies to a large extent.

For Tanzania, the study by Arndt et al. (2012) used a recursive dynamic CGE model to investigate the feasibility of biofuel production and estimate its impacts on the economy. The results showed that the engagement of smallholder farmers and improved productivity could reduce poverty in developing countries. Moreover, Flexor and Kato (2017) suggested that inclusive development and social inclusion based on biofuel policies in Brazil depended primarily on a constellation of public actions capable of creating several economic opportunities for small-scale farmers.

However, several studies also show the adverse effects of biofuel policy implementation. Gohin (2008) evaluated the effects of the EU biofuel policy on EU markets for agricultural and food products and on-farm incomes using a CGE model. The results showed that biofuel refineries, in the case of the EU, may have to rely on imports from the world market, particularly in the case of biodiesel production, which is not as highly protected (by tariffs) as bioethanol production. The new demand will be satisfied mostly by more significant domestic production (64.5%) and by the EU shift from being a net exporter to a net importer. On the other hand, the EU biofuel policy will likely strengthen the prices of arable crops, so agricultural (livestock) sectors downstream may suffer through increased production costs.

In the case of the US biofuel policy, Elobeid et al. (2006) identified adverse effects for the US livestock sector due to the significant increase in corn prices. For instance, the expected growth in arable crop production may intensify land-use competition with a possible decrease in pastureland. Tyner and Taheripour (2008) investigated the economic consequences of further expansion in the ethanol industry for the key economic variables of the US agricultural and energy markets and found that both ethanol and biodiesel production involve potentially large land-use changes globally. According to Altenburg et al. (2009), some critics claim that the production of biodiesel will lead to food scarcity and seizure of common lands by corporate investors, putting livelihoods at risk. Some also question whether the life-cycle carbon balance—i.e., the net carbon effect after taking inputs, transport, and other emission sources into account—is positive.

Despite the pros and cons of biodiesel policy implementation, according to Gunatilake et al. (2011b), the biodiesel sector also faces many challenges regarding the allocation of land to oilseed plantations; resolving property rights issues of the wastelands; developing high-yielding varieties and suitable agronomic practices; and correcting information and coordination failures that have prevented the development of markets. However, these issues may be counterbalanced by growing energy crops on land that has been set aside. Furthermore, according to Gunatilake et al. (2011a), more indirect approaches to protecting against energy price shocks can also be considered, such as promoting energy efficiency and improving agro-food productivity. Overall, previous research on biofuel policy implementation showed mixed results and has not produced any clear conclusion. This paper builds on the existing literature by investigating the economic impacts of the biodiesel sector in the case of Indonesia.

## Methodology

### Model

An Indonesian CGE model was utilized to assess the economic effects of the B30 mandate, which is expected to produce economic benefits both from macro- and micro-perspectives. The core of the model is a combination of the well-established CGE model ORANI (Horridge et al. 1999), the WAYANG (Warr 1998), and the INDOF model (Oktaviani 2001; Oktaviani et al. 2011). WAYANG is an Indonesian CGE model adapted from the ORANI^1^ CGE model by adding several blocks of equations in the model, including regional extension (Warr 1998). INDOF is an Indonesian CGE model that already included land mobility equations in the model, allowing land to be mobile across industries, and hence will adjust to the land rental price (Oktaviani 2001; Oktaviani et al. 2011). In Indonesia, land is mobile when land transformation (the utilization of land from one activity to another) occurs rapidly. To capture the land mobility phenomenon as well as, the economic impacts of biodiesel policy in Indonesia at the regional level, the study team combined WAYANG and INDOF models.

To be more specific, the combined model can be constructed at the aggregate level but is typically built with considerable micro-level detail and explicit interdependencies among the components of the economy: industries, households, investors, governments, importers and exporters, and between different markets. The organization of the model is constructed into 18 blocks, as represented in Table 1.

**Table 1:** List of equation blocks in the model

No.	Equation blocks
1	Labor demand
2	Primary factors demand
3	Intermediate inputs demand
4	A composite of intermediate inputs and primary factors
5	A composite of output by industry
6	Investment goods demand
7	Demand from households
8	Export demand
9	Margin demand
10	Prices of purchasing agents
11	Market clearing
12	Indirect taxes
13	Income and expenditure gross domestic product (GDP)
14	Balance of trade and other aggregates
15	Rates of return
16	The accumulation of investment capital
17	The accumulation of debt
18	The extension of regions

Source: Oktaviani (2001), Oktaviani et al. (2011)

**Fig. 1:**
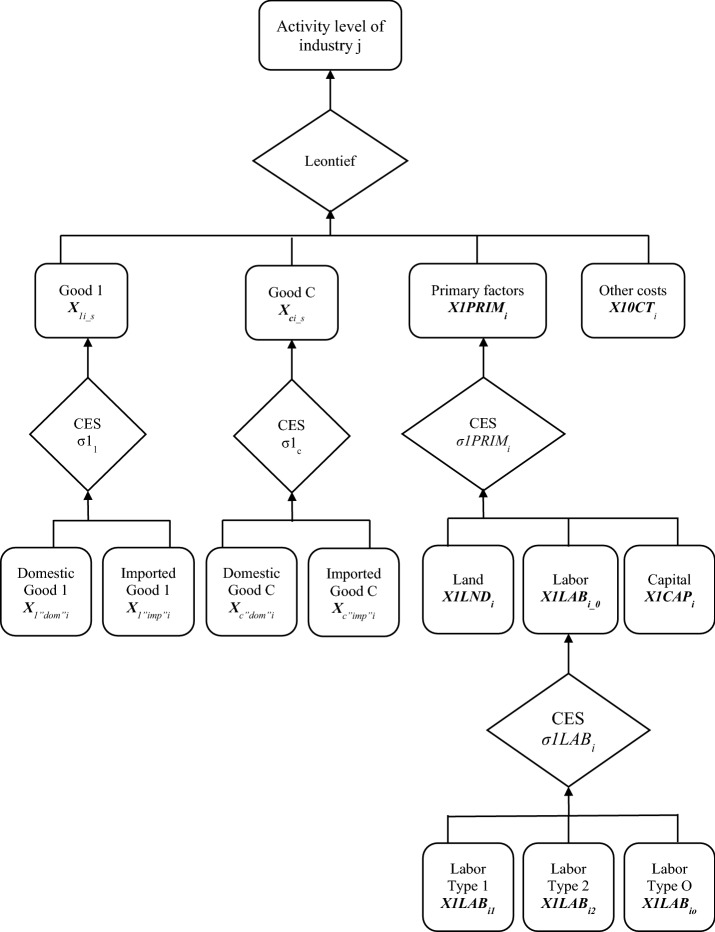
Structure of production in the Indonesian CGE model for biodiesel policy. Source: Horridge et al. (1999)

An emphasis was placed on the block regarding intermediate input as the biodiesel mandates will change the demand structure of related industries, such as oil refineries and the basic chemicals industry. The theoretical foundation of the production structure in the CGE Model explains that one industry does not correspond to one commodity, as it is able to produce a number of commodities. It also demands both intermediate and primary inputs in the form of labor and capital from the domestic market and from imports. Meanwhile, land input is sourced domestically. Constant elasticity of substitution was used in the function of production to represent assumptions of the separability of input and output in a multistage production structure. The additional assumption of Leontief technology in adopting fixed proportions was made in the composite demands of both intermediates and aggregate primary factors. There are two important behavioral properties of the production structure, assuming that agents involved in the markets are price takers and act rationally to maximize profits by using the most efficient combination of inputs according to the available level of technology. The production structure is represented in Fig. 1.

Figure 1 explains that the production function in the model was stated as:

F (input, output) = 0 in a perfectly competitive industry.

and

$${\mathrm{G}}{(\mathrm{input})} = {\mathbf{X1TOT}} = {\mathrm{H}}{(\mathrm{output})},$$

where **X1TOT** represents the activity in the industrial level index, with G and H constituting the input and transformation of output. The input–output separability was held. It has been noted that the production structure—resulting in the outputs of each industry—follows several stages of production (Fig. 1). Hence it was driven by intermediary activity at the industrial level (Blackorby et al. 1978). Meanwhile, the Leontief production function became a basis for the level of output in the industry at the composite level of commodity, primary factor, and other costs. It was assumed that the Leontief production function is described by the ratios of input combination. Consequently, the ratios and prices of inputs formed the industry level share of expenditure defining the production function:

$${X1TOT}_{i}=\frac{1}{{A1TOT}_{i}}\left.\left\{\,{{\vphantom{\frac{{X1}_{ci\_s}}{{A1}_{ci\_s}}}}_{C\in COM}^{\,\quad\,MIN}}\!\!\!\left(\frac{{X1}_{ci\_s}}{{A1}_{ci\_s}}\right),\frac{{X1PRIM}_{i}}{{A1PRIM}_{i}},\frac{{X1OCT}_{i}}{{A1OCT}_{i}} \right. \right\}i\in IND,$$

where *X1TOT*_*i*_: the activity at the industrial level in the industry *i*; *A1TOT*_*i*_: technical change for input in the industry *i; *X1_*ci_s*_: import and domestic commodity composite demand *c* in the industry *i*; A1_*ci_s*_: technical change for composite commodity *c* on both domestic market and imports; *X1PRIM*_*i*_: composite of primary factors; *A1PRIM*: technical change for primary factors; *X1OCT*_*i*_: other cost demand in industry *i*; *A1OCT*_*i*_: other costs, technical change in industry *i*; *IND*: industry.

Other costs cover various miscellaneous industry expenses that have not been covered in the expenses of primary inputs (land, labor, capital), intermediate inputs, and production taxes. Examples of the other costs in Indonesia are the expenses for business and professional licenses, stamp duty expenses, and if any, pollution and international transactions. The value of other costs varies, depending on the sector. Our calculations show that the proportion of other costs in all sectors included in the CGE model is about 0.73% of the total production costs.

The CGE model consists of a set of commodities that correspond to each industry. The commodities include 185 goods and services produced by 185 corresponding industries: 36 agricultural industries, 13 mining industries, 89 manufacturing and utility industries, and 47 services. Each commodity can be sourced either domestically or via imports.

The CGE model also covers 34 provinces in Indonesia. This study uses the top-down multi-regional approach (Oktaviani 2008, 2011). The specification of the model in this study lies in the regional extension equation. With this equation, the top-down approach to the CGE model can be captured so that it reflects the policy linkages between the national and regional performance. After constructing the national model, we use Interregional Input Output (IRIO) data to disaggregate the equation into the number of regions. The following are some additional equations needed for the development of the regional extension blocks (Oktaviani 2008), namely:

Demand for intermediate inputs by commodity, source, industry and region:

$${\text{X1}}_{{{\text{CSI}}}} \_{\text{REG}}_{{{\text{csir}}}} \, = \,X1_{\text {csi}} * \, RGSHR1_{\text {ir}} ,$$

where *X1*_csi_, intermediate input demand by commodity, source and industry; *RGSHR1*_ir_, share of input between regions based on industry and region.

Investment demand by commodity, source, industry and region.

$${\text{X2}}_{{{\text{CSI}}}} \_{\text{REG}}_{{{\text{csir}}}} \, = \,X2_{{{\text{csi}}}} *{\text{ RGSHR}}2_{\text {ir}} ,$$

where *X2*_csi_, investment demand by commodity, source and industry; RGSHR2_ir_, share of regional input investment by industry and region.

Demand for consumption of goods by commodity, source, region and household:

$${\text{X3CS}}\_{\text{REG}}_{{{\text{csrh}}}} \, = \,X3_{{{\text{csh}}}} *{\text{ RGSHR}}3_{{{\text{cr}}}} ,$$

where *X3*_csh_, demand for consumption by commodity, source and household; RGSHR3_cr_, share of regional consumption demand by commodity and region.

Export demand by region:

$${\text{X4}}\_{\text{REG}}_{{{\text{cr}}}} \, = \,X4_{\text c} * \, RGSHR4_{\text {cr}} ,$$

where *X4*_c_, export demand by commodity; RGSHR4_cr_, share of exports by commodity and region.

This model also has a feature of eight representative households. They consist of five rural and three urban household groups, where: (i) Rural 1 is agricultural workers; (ii) Rural 2 refers to agricultural entrepreneurs; (iii) Rural 3 is low-income non-agricultural households in rural areas, namely low-income entrepreneurs, administrative staff, mobile traders, casual workers in the transportation sector, individual services, and unskilled laborers; (iv) Rural 4 is the non-labor force in rural areas, which includes undefined groups in rural areas; (v) Rural 5 is non-agricultural upper-class households in rural areas, including upper-class entrepreneurs, non-agricultural entrepreneurs, managers, military personnel, professionals, technicians, teachers, upper-class administrative workers and salespeople; (vi) Urban 1 is a non-agricultural household of the lower class in urban areas, which includes low-income independent entrepreneurs, administrative staff, mobile traders, casual workers in the transportation sector, individual services and unskilled laborers; (vii) Urban 2 is the non-labor force in urban areas, including undefined groups.; and (viii) Urban 3 are non-agricultural households in the upper income class, such as independent entrepreneurs, non-agricultural entrepreneurs, managers, military personnel, professionals, technicians, teachers, administrative staff, and upper-income-class salespeople.

In addition, labor as a primary factor is disaggregated into nine types: managers, professionals, technicians, administrators, salespeople, skilled labor in agriculture, skilled labor in manufacturing, operators, and unskilled labor.

Meanwhile, some information on the important behavioral parameters consisting of Armington elasticity, primary input elasticity, labor elasticity, expenditure elasticity, and export demand elasticity is presented in Table 2. It can be identified that the elasticities for Armington, labor, and export demand are identical. Therefore, the uniform value of elasticities across sectors is considered one of the limitations of this study. Next, to test the robustness of the results related to the utilization of the elasticities, the study team conducted a sensitivity analysis.

**Table 2:** Elasticities in the model

Sector	Armington Elasticity	Primary input elasticity	Labor elasticity	Export demand elasticity
Basic chemicals	2	1.21	0.5	− 0.5
Non-metal minerals	2	1.21	0.5	− 0.5
Chemical products	2	1.21	0.5	− 0.5
Sugar	2	1.21	0.5	− 0.5
Sugarcane	2	1.21	0.5	− 0.5
Varnish and lacquer	2	1.21	0.5	− 0.5
Cosmetics	2	1.21	0.5	− 0.5
Soap	2	1.21	0.5	− 0.5
Pharmaceutical products	2	1.21	0.5	− 0.5
Oil	2	0.61	0.5	− 0.5
Vegetable oils	2	1.21	0.5	− 0.5
Palm oil	2	1.21	0.5	− 0.5
Other cereals	2	0.71	0.5	− 0.5
Soybean	2	0.71	0.5	− 0.5
Vegetables	2	0.71	0.5	− 0.5
Fruits	2	0.71	0.5	− 0.5
Oil refineries	2	1.21	0.5	− 0.5
Maize	2	0.71	0.5	− 0.5

Source: Oktaviani (2001), Oktaviani et al. (2011)

The impact of biodiesel mandates is assessed using long-run closure where capital is assumed to be mobile between sectors, and the rate of return is determined by the global rate of return. On the macro-level, the closure also satisfies the external balance between capital and current accounts. Real government consumption is an exogenous variable, while household consumption is treated as an endogenous variable. The trade balance influences the real exchange rate. The industry outputs are set as endogenous variables, allowing the study team to analyze the impact of shocks on the output of industries.

### Data

To construct a CGE model for biodiesel policy, we collected several data including the Indonesian input–output (I–O) table and other behavioral parameters (e.g., Armington elasticity, input elasticity). The latest I–O table was published by the Statistics Indonesia agency in 2021 (BPS 2021a) to capture the state of the Indonesian economy in 2016. For the regional aspect, we utilized the IRIO data produced by Statistics Indonesia in 2021, capturing 34 provincial interlinkages in Indonesia in 2016 (BPS 2021b).

**Fig. 2:**
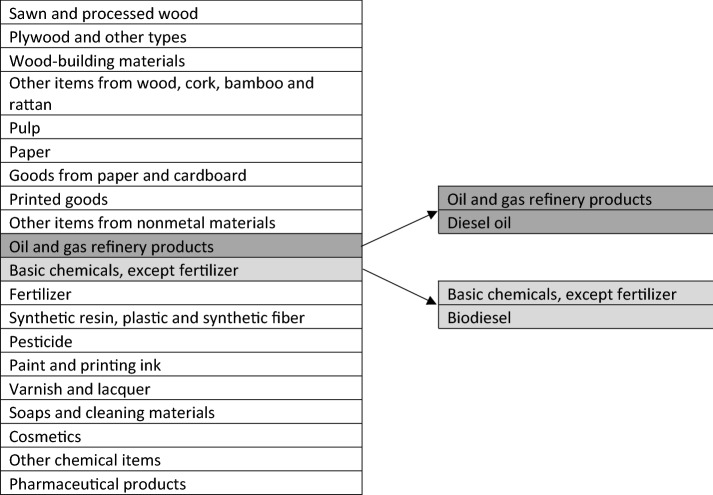
Biodiesel industry in the 2016 Indonesian I–O table

Despite its relatively extensive sector aggregation—185 commodities—the discussion at sectoral level focuses on several sectors, particularly the upstream and downstream biodiesel-related sectors. It is important to note that no biodiesel industry is included in the I–O table. However, according to the Indonesian Ministry of Industry, KBLI^2^ (Version 2009) sub-class 20115 is part of KBKI^3^ (Version 2010) sub-class 345^4^ in the manufacture of basic chemical products. In practice, biodiesel is not used directly either as an intermediate input or as energy for production. It is normally mixed with diesel fuel to satisfy B30 regulations. Technically, diesel fuel is a subset of oil and gas refinery products. Based on the most updated Indonesian I–O table and Energy Balances of Indonesia for 2019 (BPS 2020), the share of automotive diesel oil in the refinery industry is 11% and the share of biodiesel in the basic chemicals industry is 7.48%. As such, we use these two sectors to represent the biodiesel industry (Fig. 2).

### Policy simulations

Since all equations in the CGE model are stated in percentage changes instead of levels of economic variables, the policy simulations are formulated in percentage form. Adding in the Indonesian context related to the implementation of the biodiesel mandate, the study team included several stages to set up the magnitudes in each policy simulation.

The magnitude of shocks in the scenarios was calculated by using the share of automotive diesel oil in the refinery industry, the share of biodiesel in automotive diesel oil, and the share of biodiesel in the basic chemicals industry. The current share of biodiesel in automotive diesel oil is taken from the Minister of Energy and Mineral Resources Decree No. 25 of 2013—this figure is 5%. As outlined previously, the shares of automotive diesel oil in the refinery industry and in the basic chemicals industry are 11% and 7.48%, respectively.

For the default condition (2016), the share of biodiesel in automotive diesel oil is 5% (or equal to 7.48% of the total output of the basic chemicals industry), while the implementation of B30 requires the share of biodiesel to increase to 25% higher than the default condition. Consequently, the output of the basic chemicals industry rises as much as (0.0748 * (1 + 0.25)) * 100 = 9.35%. The changes in output in the oil and gas refinery industry are calculated by assuming that a higher share of biodiesel will reduce the share of automotive diesel oil. Thus, the output of the oil and gas refinery industry will be corrected by as much as ((0.11 * 0.05 * 1) − (0.11 * 0.05 * 0.70)) * 100 = 0.165%.

An external challenge considered in this study is the decision by the EU to ban the use of biodiesel in motor fuels from 2021 to prevent deforestation and attain climate-related goals. Banned products include biodiesel from Indonesia and Malaysia. Here, we estimate the magnitude of policy shock from the EU's ban on biodiesel imports from Indonesia. The ratio of palm oil exports to Indonesia’s total exports in 2020 was 10.63%.^5^ The Indonesian share of exports to the EU as a percentage of exports to the world market in 2020 was 12.95% (ITC 2021), and 40% of Indonesian palm oil exports to Europe are processed into biodiesel (Soeriatmaja and Leong 2018), so we estimate the shock to be − 0.1295*0.4*1. Hence, the policy shock from the biodiesel import ban is − 5.18%. This shock was used to represent the combined shock from the B30 mandate. We also identify that the consequences of the import ban will increase the domestic availability of vegetable oils as input for the biodiesel industry in Indonesia. The B30 mandate policy is assumed to fully absorb the oversupply of Crude Palm Oil (CPO) for domestic use. This claim is also supported by the fact that there was no significant change in the composition of the market destination of CPO exports due to the implementation of the B30 mandate. The market composition remained the same in 2017 and 2020 as India, China and the European Union ranked as the top three markets for Indonesia^6^ (ITC Trade Map 2022b). Based on the latest I-O table, the share of demand for vegetable oils in the chemicals industry is 0.09. Hence, the magnitude of increasing output in the basic chemicals industry equals (0.05 * 0.09) * 100 = 0.45%. We add this to the existing B30 basic chemicals industry shock and get 10.19%. Using the same database, we also estimate how much the vegetable oils sector will respond. Based on the I–O table, the vegetable oils sector predominantly uses intermediate inputs from its own sector. The share of the use of vegetable oils is 0.6. Hence, the magnitude of augmenting output in this sector is (0.05 * 0.6) * 100 = 3.00%.

In short, we conducted two simulations:

- Simulation 1: Fulfillment of 30% biodiesel blending target (B30 mandate) by increasing the output of the basic chemicals industry by 9.35% and by decreasing the output of oil and gas refining by 0.165%.
- Simulation 2: Fulfillment of 30% biodiesel blending target (B30 mandate) by increasing the output of the basic chemicals industry by 10.19%, increasing the output of vegetable oils by 3.00%, and decreasing the output of oil and gas refining by 0.165%, while the EU's biodiesel trade ban led to an export decline of 5.18%.

Technically, to simulate B30 mandates, we set the general tax shifter to be endogenously determined so that we could adjust the output of basic chemicals, oil refineries, and vegetable oils. Meanwhile, we approached the simulation of the EU ban on vegetable oils by decreasing the export demand shifter of Indonesian vegetable oils.

## Results and discussion

The impact of the biodiesel mandate—due to the fulfillment of the biodiesel blending target and the EU's biodiesel trade ban—on macroeconomic variables is presented in Table 3. The fulfillment of the 30% biodiesel blending target (Simulation 1) will increase the performance of real GDP by 0.058% and produce a trade surplus (0.023%). A decrease in trade is followed by an increase in real household consumption (0.018%). The intensive use of biofuels will reduce consumer dependency on fossil fuels through higher substitutability between fossil fuels and biodiesel. The combination of the two effects will relieve pressures on inflation from fuel commodities because Indonesia is no longer considered a net exporter of petroleum (Oktaviani et al. 2011). Moreover, implementing the biodiesel mandate increases the average real wage by 0.096%.

**Table 3:** Biodiesel policy impact on Indonesian macroeconomic performance

Macroeconomic variables	Percentage change (%)	
	Simulation 1	Simulation 2
Balance of trade/GDP	0.023	− 0.122
Terms of trade	− 0.127	− 0.734
Average capital rental	0.004	− 0.112
Consumer price index	0.026	0.120
Average real wage	0.096	0.200
Real GDP from expenditure side	0.058	0.103
Real household consumption	0.018	0.151
Import volume index	− 0.195	− 0.196
Aggregate real investment expenditure	0.017	0.145
Export volume index	0.064	− 0.105
Aggregate real government demand	0.038	0.211

Source: Author’s calculation

The external challenges of biodiesel development were also simulated by assuming that the EU's biodiesel trade ban will result in decreased Indonesian exports. Considering the trade ban and the fulfillment of the 30% blending target (Simulation 2) surprisingly produces a better economic impact than the B30 mandate (Simulation 1). The fulfillment of the 30% blending target will increase real GDP to 0.103% even though it will also worsen the trade balance to 0.122% (from 0.023%), where both export and import volumes are decreasing. The results also highlight that the B30 mandate and the combined effect of the EU trade ban still yield an inflationary effect in the short term due to the ample demand. However, it potentially raises the average real wage to 0.135% (from 0.096%).

The B30 mandatory implementation policy, or mixing 30% diesel oil with 30% biodiesel as part of efforts to overcome the current account deficit, will be adopted by various sectors in Indonesia. The results in Table 4 indicate that the B30 mandate is expected to incentivize local sales of the basic chemicals sector by 4.414%, constituting the highest response in sales increase. This significant rise in basic chemical sales is due to increased productivity, as the component fatty acid methyl esters (FAME) sourced from crude palm oil is produced in this sector.

**Table 4:** Impacts of biodiesel mandates on biodiesel local sales and related sectors in Indonesia%

Sector	Simulation 1	Simulation 2
Basic chemicals	4.414	5.285
Non-metal minerals	1.028	1.224
Chemical products	0.402	0.402
Sugar	0.340	0.446
Sugarcane	0.326	0.429
Varnish and lacquer	0.323	1.509
Cosmetics	0.241	0.684
Soap	0.188	0.790
Pharmaceutical products	0.122	0.166
Oil	0.111	0.120
Vegetable oils	0.100	0.532
Palm oil	0.086	0.451
Other cereals	− 0.077	− 0.311
Soybean	− 0.073	− 0.284
Vegetables	− 0.014	0.002
Fruits	− 0.014	− 0.005
Oil refineries	− 0.014	− 0.005
Maize	− 0.003	− 0.010

Source: Author’s calculation

Aside from supporting national energy security, the biodiesel mandate is also expected to increase the added value of palm oil downstream industries. Based on the results of the data processing, some sectors benefit from the implementation of B30. Several sectors downstream of crude palm oil—such as soap and cleaning agents, synthetic resins, and cosmetics—will also show a significant increase in local sales compared with other sectors. The animal-based and vegetable oil sectors tend to respond positively and increase sales after implementing the B30 mandate, despite its relatively small effect (0.100%). The results also indicate an improvement in the palm oil sector, as the B30 mandate increases its local sales by 0.451%. Meanwhile, the related local sales of the sectors with the lowest decline, as expected, are oil and gas refineries by 0.014%. The results also validated that several agricultural sectors—such as other cereals, soybeans, vegetables, fruits, and maize—showed negative responses. The sectors experienced declining local sales as resources are reallocated to the sectors incentivized by the mandates (e.g., oil palm, animal-based and vegetable oils, and basic chemicals).

The result of the simulation combining the B30 mandate (from the side of domestic policy) with the EU's biofuel-based trade ban (in terms of international trade policy) is presented in Table 4. The result shows significant progress in two main sectors. The local sales from the basic chemicals sector increase by 5.285%, while the local sales from the vegetable oil sector (including crude palm oil) rise by 0.532%. The combination of the B30 mandate and the EU ban is expected to favor the domestic utilization of crude palm oil in Indonesia. Therefore, the increasing supply of crude palm oils in the domestic market will be fully absorbed to support the biodiesel industries. The historical biodiesel production data also confirmed close substitution between the export subject to the EU's ban and the domestic absorption of CPO. The initiation of the EU renewable energy directive (RED II) against crude palm oil (CPO) in 2018 has incentivized domestic utilization of CPO in the biodiesel industry. The realization of biodiesel production jumped from 6168 thousand kiloliters in 2018 to 8399 thousand kiloliters in 2019. This positive trend has persisted and reached 10,240 thousand kiloliters in 2021 (MEMR 2021). Positive developments in the vegetable oil and basic chemicals sectors will be followed by a sales increase in derivative products—which have a strong forward linkage with crude palm oil and biodiesel—such as soap and cleaning materials, synthetic resins, plastics, synthetic fibers, and cosmetics. On the other hand, sales of the oil and gas refining sector are expected to fall by approximately − 0.005%, as the resources are mobilized for the biodiesel and related sectors.

The impact of the biodiesel mandate due to the fulfillment of the biodiesel blending target and the EU's biodiesel trade ban on sectoral prices is presented in Table 5. The impacts on sectoral prices reveal an identical pattern both in Simulation 1 (B30 mandate) and Simulation 2 (B30 mandate plus trade ban). Developing biodiesel has led to a decline in output prices for the basic chemicals sector by − 5.435% and − 6.461%, respectively. In the meantime, prices in the vegetable oils sector (crude palm oil) rose by 0.32%. This outcome is in line with the previous predictions of the Indonesian Palm Oil Association, which stated that this policy could promote an increase in crude palm oil prices to as much as USD 50 per ton (Gumelar 2018). This is mainly due to the demand for crude palm oil in the domestic market, increased with the implementation of the B30 mandate and absorbed by the basic chemicals industry.

The additional scenario of the EU trade ban will drive an oversupply of crude palm oil in the domestic market and potentially decrease the price by − 6.83%. The output prices of oil and gas refineries in Simulation 1 and Simulation 2 rise by 0.66% and 0.82%, respectively. Reducing the composition of diesel fuel in the energy mix potentially restricts its production, thereby increasing the output price.

**Fig. 3:**
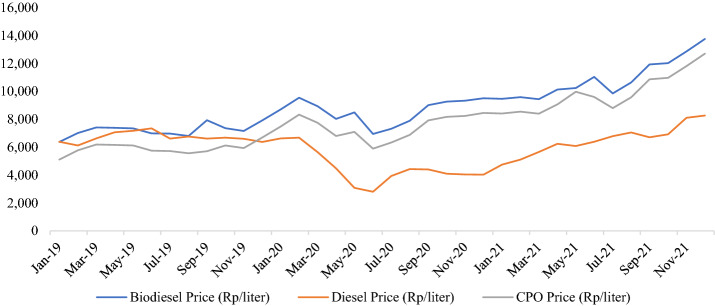
Prices of crude palm oil, biodiesel and diesel oil (January 2019–December 2021). Source: Ministry of Energy, Mineral and Resources (2021b, 2021c)

Figure 3 shows the historical prices of crude palm oil, biodiesel, and diesel oil for 2019–2021. Since the enactment of the B30 mandate in Indonesia, the prices of crude palm oil and biodiesel have decreased, particularly in 2019. This indicates that the implementation of the mandate has incentivized the absorption of crude palm oil in the biodiesel industries. It also increases prices at the farm-gate level, as reflected in the expectation that the price of palm oil will rise by 0.17% and 0.72% in Simulations 1 and 2. Several agricultural sectors—such as sugarcane, fruits, vegetables, soybeans, other cereals, and rice—are among the losing sectors due to land conversion leading to higher food prices.

In Simulation 2, the price of vegetable oils fell by 6.830% (Table 5). Without market diversification, the EU trade ban could redirect crude palm oil to the domestic market, causing overall prices to decline. Indonesia's crude palm oil exports to the EU reached 1.58 million tons in January–April 2018. On the other hand, the decline in prices for animal-based and vegetable oils also affects the price of derivative products. For example, the price of soap and cleaning materials in Simulation 2 decline by 1.581%. In connection with the B30 mandate and the trade ban, the prices of oil and gas refining still increase by 0.820% and 1.017%, respectively.

**Table 5:** Impacts of biodiesel mandates on biodiesel prices and related sectors in Indonesia

Sector	Simulation 1	Simulation 2
Basic chemical	− 5.435	− 6.461
Cosmetics	− 0.462	− 1.048
Chemical products	− 0.448	− 0.516
Soap	− 0.411	− 1.581
Sugar	− 0.388	− 0.322
Varnish and lacquer	− 0.301	− 1.451
Pharmaceutical products	− 0.082	0.040
Other cereals	0.069	0.292
Soybean	0.074	0.317
Oil	0.106	0.212
Vegetables	0.111	0.517
Fruits	0.117	0.547
Maize	0.137	0.551
Palm oil	0.170	0.723
Sugarcane	0.301	0.715
Vegetable oils	0.322	− 6.830
Non-metal minerals	0.505	0.696
Oil refineries	0.663	0.820

Source: Author’s calculation

The trade-related impacts of the policies are presented in Table 6. As the local sales of the basic chemicals sector increase in all simulations, it will potentially raise exports and decrease imports. The B30 mandate also benefits other sectors with strong links to basic chemicals, such as animal-based and vegetable oils, soap products, and cosmetics. The results in local sales and the trade balance for synthetic resins, soap and cosmetics show some improvement. Meanwhile, in the animal-based and vegetable oils sector, exports decreased in Simulations 1 and 2 by 0.161% and 1.585%, respectively.

**Table 6:** Impacts of biodiesel mandates and trade ban on biodiesel trade and related sectors in Indonesia

Sector	Sim 1		Sim 2	
	Export	Import	Export	Import
Basic chemical	2.718	− 4.203	3.231	− 4.918
Cosmetics	0.231	− 0.681	0.524	− 1.401
Chemical products	0.224	− 0.365	0.258	− 0.402
Soap	0.205	− 0.636	0.791	− 2.371
Sugar	0.194	− 0.381	0.161	− 0.148
Varnish and lacquer	0.151	− 0.030	0.726	− 0.255
Pharmaceutical products	0.084	− 0.201	0.047	0.002
Other cereals	− 0.012	0.069	− 0.076	0.458
Soybean	− 0.037	0.073	− 0.159	0.344
Oil refineries	− 0.053	0.199	− 0.106	0.427
Vegetables	− 0.055	0.206	− 0.258	1.032
Fruits	− 0.059	0.210	− 0.273	1.027
Maize	− 0.069	0.271	− 0.276	1.093
Palm oil	− 0.085	0.399	− 0.362	1.782
Sugarcane	− 0.150	0.934	− 0.357	1.866
Vegetable oils	− 0.161	0.539	− 1.585	− 9.071
Non-metal minerals	− 0.253	2.295	− 0.348	2.920
Oil	− 0.331	1.660	− 0.410	2.027

Source: Author’s calculation

Attention should be focused on the B30 mandate's impacts on imports in the oil and gas refining and oil sectors. Despite the mandate, the current biodiesel policy will not automatically improve the trade balance for oil refining, as imports are still expected to rise in all simulations. However, the magnitudes are relatively small, ranging from 0.199 to 0.427%.

At the regional/subnational level, it can be seen that all simulations yield positive impacts on the gross regional domestic product (GRDP) in each province of Indonesia (Table 7). GRDP in some provinces has increased more than in others. In Central Kalimantan, Riau, South Sumatra, and East Kalimantan, for example, GRDP will experience a large increase due to the implementation of the B30 mandate. This is because Riau Province, a major palm oil producer in Indonesia, has benefited more from this policy.

**Table 7:** Impacts of biodiesel mandates and trade ban on regional gross domestic product

Rank	Province	Simulation 1	Simulation 2
1	Central Kalimantan	0.074	0.145
2	East Java	0.082	0.145
3	Riau Islands	0.087	0.141
4	South Sumatra	0.091	0.138
5	Riau	0.061	0.138
6	East Kalimantan	0.094	0.137
7	Central Java	0.076	0.132
8	Jambi	0.067	0.129
9	West Java	0.085	0.124
10	West Sulawesi	0.049	0.120
11	Bengkulu	0.060	0.114
12	Central Sulawesi	0.073	0.109
13	Bangka Belitung	0.070	0.107
14	Lampung	0.053	0.106
15	West Kalimantan	0.047	0.102
16	South Kalimantan	0.053	0.101
17	Bali	0.048	0.097
18	West Papua	0.064	0.097
19	Special Province Yogyakarta	0.047	0.094
20	Southeast Sulawesi	0.053	0.089
21	North Sumatra	0.032	0.088
22	Aceh	0.046	0.088
23	Banten	0.047	0.083
24	Gorontalo	0.039	0.083
25	West Sumatera	0.038	0.081
26	West Nusa Tenggara	0.042	0.080
27	East Nusa Tenggara	0.038	0.079
28	South Sulawesi	0.036	0.076
29	Papua	0.045	0.073
30	North Maluku	0.038	0.072
31	North Sulawesi	0.031	0.070
32	North Kalimantan	0.023	0.061
33	Maluku	0.025	0.058
34	Special Province DKI Jakarta	0.023	0.048

Source: Author’s calculation

The provinces of East Java, Central Java and West Java have also benefited from the B30 mandate, demonstrating that downstream industries related to oil palm and basic chemicals were mostly located in these provinces. For example, basic chemicals industries are mostly located on the island of Java (Jakarta, Banten, West Java and East Java), not in oil palm-producing areas such as Central Kalimantan, Riau, South Sumatra and East Kalimantan.

The impact of biodiesel mandates (and trade ban) on real household income is presented in Table 8. The biofuel mandates cause positive impacts on poor rural and urban households in Indonesia. Household groups of Rural 1 and Urban 3 benefit most, both in Simulation 1 and 2. The majority of farmers who are classified in Rural 1 households show the highest increase in real income due to expansion of the animal-based and vegetable oils sector and the oil palm sector. Meanwhile, the richest household group in urban areas (Urban 3) significantly benefited as the price of biodiesel in the basic chemicals sector decreased significantly.

**Table 8:** Impacts of biodiesel mandates and trade ban on real household income

Household	Simulation 1	Simulation 2
Rural 1	0.080	0.170
Rural 2	0.077	0.162
Rural 3	0.076	0.158
Rural 4	0.076	0.159
Rural 5	0.036	0.020
Urban 1	0.074	0.153
Urban 2	0.055	0.087
Urban 3	0.080	0.184

Source: Author’s calculation

An additional sensitivity analysis is performed to ensure that changes in elasticities will not significantly change the magnitude of the results. We performed the sensitivity analysis by setting arbitrary 5% increases in several behavioral parameters, namely: Armington elasticity, primary input elasticity, labor elasticity, and export demand elasticity. The sensitivity analysis results on the macroeconomic level (Table 9) show that reducing the elasticities of basic chemicals and oil refineries by half and doubling up the parameters produce insignificant differences. The signs (positive or negative) of the results are also consistent. Therefore, we can infer that our findings are robust.

**Table 9:** Results sensitivity analysis: reducing the parameters by half and doubling up the parameters

Macroeconomic variables	Result of simulation (%)		Result of sensitivity analysis where parameters are reduced by half (%)		Result of sensitivity analysis where parameters are doubled (%)	
	Simulation 1	Simulation 2	Simulation 1	Simulation 2	Simulation 1	Simulation 2
Balance of trade/GDP	0.023	− 0.122	0.005	− 0.151	0.048	− 0.092
Terms of trade	− 0.127	− 0.734	− 0.179	− 0.752	− 0.094	− 0.660
Average capital rental	0.004	− 0.112	− 0.029	− 0.139	0.028	− 0.077
Consumer price index	0.026	0.120	0.041	0.130	0.016	0.103
Average real wage	0.096	0.200	0.112	0.206	0.084	0.176
Real GDP from expenditure side	0.058	0.103	0.058	0.099	0.059	0.100
Real household consumption	0.018	0.151	0.050	0.186	− 0.014	0.116
Import volume index	− 0.195	− 0.196	− 0.114	− 0.094	− 0.276	− 0.273
Aggregate real investment expenditure	0.017	0.145	0.017	0.137	0.018	0.138
Export volume index	0.064	− 0.105	0.043	− 0.141	0.084	− 0.093
Aggregate real government demand	0.038	0.211	0.048	0.212	0.030	0.192

Source: Author’s calculation

## Conclusion and policy implications

This paper used a CGE simulation to examine the impact of 30% biodiesel blending targets combined with the EU's import ban on biodiesel products. The results show that two simulations positively impact macroeconomic variables, including real GDP, real wages, and real household consumption.

The sectoral impacts show that the B30 mandate increases the local sales of the basic chemicals sector and other sectors related to crude palm oil and biodiesel, such as soap and cleaning agents, synthetic resins, and cosmetics. The implementation of a trade ban will further incentivize domestic biodiesel production due to the increased availability of crude palm oil. It will also drive higher output in the oil palm and downstream sectors. Meanwhile, sales of the oil and gas refining sector will decline since resources are expected to be mobilized for biodiesel and related sectors.

The impacts on sectoral prices show identical patterns in Simulation 1 and Simulation 2. Both indicate a decline in output prices for basic chemicals and an increase in output prices for oil and gas refining. The trade implications of the B30 mandate will lead to an improvement in the trade balance of the basic chemicals and downstream sectors. However, the mandate produces conflicting results for the animal-based and vegetable oils sector as exports will decline. It is also worth noting that fulfilling the 30% biodiesel blending targets will not automatically improve the trade balance of the oil and gas refining and oil sectors, since imports of these sectors are still increasing. However, the magnitudes are relatively small. As such, the GoI needs to combine its biodiesel policy with other policies, such as efficiency in oil and gas consumption at household and industrial levels. Another factor to be considered is the trade-off between food and fuels. Several agricultural sectors—such as sugarcane, fruits, vegetables, soybeans, other cereals, and rice—were also identified as losing sectors due to land conversion, leading to higher food prices.

At the regional/subnational level, the B30 mandate improves the GRDP of all provinces in Indonesia. However, oil palm-producing regions (Central Kalimantan, Riau, South Sumatra, and East Kalimantan), as well as the provinces that have strong ties to vegetable oil production and that host basic chemicals industries (East Java, Central Java and West Java) yield higher benefits than other provinces. It is also noted that the biofuel mandates result in an inclusive growth effect on rural and urban households in Indonesia.

## Data Availability

The datasets used and/or analyzed during the current study are available from the corresponding author upon reasonable request.

## Abbreviations

CGE: Computable General Equilibrium
CPO: Crude palm oil
EU: European Union
FAME: Fatty acid methyl esters
GDP: Gross Domestic Product
GHG: Greenhouse gases
I–O: Input–Output
IRIO: Interregional Input–Output
ISIC: Indonesian Standard of Industrial Classification, KBLI
ISCC: Indonesian Standard of Commodities Classification, KBKI
KBKI: Klasifikasi Baku Komoditas Indonesia, ISCC
KBLI: Klasifikasi Baku Lapangan Usaha Indonesia, ISIC
MEMR: Ministry of Energy and Mineral Resources
MT: Ministry of Trade
PSO: Public Service Obligation
RED: Renewable Energy Directive
US: United States
USD: US dollar
USDA: US Department of Agriculture

## Footnotes

1 ORANI is a CGE model of the Australian economy.

2 KBLI or *Klasifikasi Baku Lapangan Usaha Indonesia* (Indonesian Standard Industrial Classification) is a standard classification for all economic activities in Indonesia. KBLI refers to the International Standard Industrial Classification (ISIC).

3 KBKI or *Klasifikasi Baku Komoditas Indonesia* (Indonesian Standard Commodities Classification) is a standard classification for all production outputs in Indonesia up to 10-digit levels. KBKI refers to Central Product Classification (CPC).

4 The specific description for the sub-class 345 refers to Animal or vegetable fats and oils and their fractions, chemically modified, except those hydrogenated, inter-esterified, re-esterified or dielaidinized; inedible mixtures or preparations of animal or vegetable fats or oils.

5 [ITC] International Trade Center (2022a) Export of Indonesian CPO to world in 2020. Trade map: Trade statistics for international business development. Available at https://www.trademap.org/Country_SelProduct_TS.aspx?nvpm=1%7c%7c42%7c%7c%7c1511%7c%7c%7c4%7c1%7c1%7c2%7c2%7c1%7c3%7c1%7c1%7c1. Accessed 1 June 2022.

6 [ITC] International Trade Center (2022b) Market positioning of Indonesian CPO exports 2017 and 2020. Trade map: Trade statistics for international business development. Available at https://www.trademap.org/Country_SelProduct_TS.aspx?nvpm=1%7c%7c42%7c%7c%7c1511%7c%7c%7c4%7c1%7c1%7c2%7c2%7c1%7c3%7c1%7c1%7c1. Accessed 1 June 2022.
